# A Microchip CD4 Counting Method for HIV Monitoring in Resource-Poor Settings

**DOI:** 10.1371/journal.pmed.0020182

**Published:** 2005-07-19

**Authors:** William R Rodriguez, Nicolaos Christodoulides, Pierre N Floriano, Susan Graham, Sanghamitra Mohanty, Meredith Dixon, Mina Hsiang, Trevor Peter, Shabnam Zavahir, Ibou Thior, Dwight Romanovicz, Bruce Bernard, Adrian P Goodey, Bruce D Walker, John T McDevitt

**Affiliations:** **1** Partners AIDS Research Center, Massachusetts General Hospital, Charlestown, Massachusetts, United States of America,; **2** Division of AIDS, Harvard Medical School, Boston, Massachusetts, United States of America,; **3** Brigham and Women's Hospital, Boston, Massachusetts, United States of America,; **4** Department of Chemistry and Biochemistry, University of Texas, Austin, Texas, United States of America,; **5** Botswana–Harvard AIDS Institute Partnership, Princess Marina Hospital, Gaborone, Botswana; Rosetta GenomicsIsrael

## Abstract

**Background:**

More than 35 million people in developing countries are living with HIV infection. An enormous global effort is now underway to bring antiretroviral treatment to at least 3 million of those infected. While drug prices have dropped considerably, the cost and technical complexity of laboratory tests essential for the management of HIV disease, such as CD4 cell counts, remain prohibitive. New, simple, and affordable methods for measuring CD4 cells that can be implemented in resource-scarce settings are urgently needed.

**Methods and Findings:**

Here we describe the development of a prototype for a simple, rapid, and affordable method for counting CD4 lymphocytes. Microliter volumes of blood without further sample preparation are stained with fluorescent antibodies, captured on a membrane within a miniaturized flow cell and imaged through microscope optics with the type of charge-coupled device developed for digital camera technology. An associated computer algorithm converts the raw digital image into absolute CD4 counts and CD4 percentages in real time. The accuracy of this prototype system was validated through testing in the United States and Botswana, and showed close agreement with standard flow cytometry (*r* = 0.95) over a range of absolute CD4 counts, and the ability to discriminate clinically relevant CD4 count thresholds with high sensitivity and specificity.

**Conclusion:**

Advances in the adaptation of new technologies to biomedical detection systems, such as the one described here, promise to make complex diagnostics for HIV and other infectious diseases a practical global reality.

## Abstract 2

A rapid affordable method for counting CD4 cells may be useful for management of HIV in resource poor settings.

## Introduction

More than 35 million HIV-infected people live in developing countries with significant resource limitations. Although 6 million people living in developing countries are in urgent need of antiretroviral therapy, only 700,000 currently receive effective treatment [1]. Global treatment efforts, including the World Health Organization's “3 by 5” Initiative, aim to extend therapy to several million people over the next few years [2]. While the cost of antiretroviral medications has dropped considerably, other obstacles, including the cost, technical, and operational requirements of CD4 counts, viral loads, and other sophisticated diagnostic tests used to initiate and monitor HIV treatment, remain to be addressed.

In particular, measurements of CD4^+^ T lymphocytes are essential for staging HIV-infected patients, determining their need for antiretroviral medications, and monitoring the course of their infection [3]. The CD4 count—expressed in adults as the absolute number of CD4 cells per microliter of blood, and in children as a percentage of total lymphocytes or total T lymphocytes—has enormous prognostic and therapeutic implications, and forms the basis for most HIV treatment decisions [4–6]. In developed countries, CD4 counts are typically performed every three to six months for each patient using the method of flow cytometry. Flow cytometers use lasers to excite fluorescent antibody probes specific for CD4 and other cell surface markers, to distinguish one type of lymphocyte from another. Several factors—including the cost of a flow cytometer (which ranges from $30,000 to $150,000), technical and operational complexity, the need for reliable electricity, and the high cost of reagents—have made these instruments impractical and/or difficult to sustain in resource-scarce settings. The urgent need for affordable and technically simple CD4 diagnostics is widely recognized [7–11].

Several efforts have been made to develop alternative, affordable CD4 counting methods for resource-poor settings. Single-purpose flow cytometers have been designed solely for counting CD4 cells, such as the Becton Dickinson FACSCount, the Partec CyFlow, and desktop instruments from Guava and PointCare Technologies. Although these newer versions make flow cytometry more affordable in some settings, reagent costs remain high, and the instruments remain expensive and in most cases, technically complex [7–13]. Low-cost microbead separation of CD4 cells from other blood cells, followed by standard manual cell counting techniques using a light microscope, offers significantly lower reagent costs than flow cytometry. These methods, however, are low throughput and extremely labor intensive, and appear to be less accurate than traditional flow cytometry; thus, they have not been widely adopted [13–18].

Less expensive CD4 counting methods that capitalize on low-cost microfabrication, efficient light sources, and affordable microelectronics and digital imaging hardware have been conceptualized, but never realized [19,20]. One of us (JTM) has previously reported the development of a novel microchip-based detection system for measuring analytes such as acids, bases, electrolytes, and proteins in solution phase [21–23]. This electronic taste chip (ETC) system carries out chemical and immunological reactions on microspheres positioned in the inverted pyramidal microchamber wells of a silicon or plastic microchip, which is housed in a miniature flow cell. Microfluidic channels deliver a series of small-volume reagents and washes to the flow cell, and hence to the chip and to each one of the microspheres. Optical signals generated by the reactions on the microspheres are visualized and captured on a charge-coupled device (CCD) with the use of transfer optics and a digital video chip. Using the ETC system, complex immunological assays, such as the ones developed to quantify cardiac risk factors in serum, can be performed with small sample volumes, short analysis times, and markedly reduced reagent costs [22].

Further development of the ETC system has shown that it could be adapted to the detection of bacteria, spores, and living cells [24]. We hypothesized that additional modifications could be made to provide accurate, low-cost CD4 counts to monitor HIV infection in resource-constrained settings. We show that a microchip-based system can perform CD4 counts from 16.5 μl of whole blood rapidly, simply, and with a high degree of accuracy compared to flow cytometry, particularly for patients with CD4 counts below 500 cells/μl. We suggest how this prototype system can be readily developed as a low-cost, portable device for use in resource-poor settings.

## Methods

### Flow Cell

The ETC system was originally designed for microsphere-based assays [21–23]. The modified version of the flow cell (see Figure 1) is enclosed within a three-piece metal casing with a flat platform permanently affixed to a circular vertical support, which is in turn connected to a screw-on cap. Within the metal casing there are top and bottom plastic inserts made from PMMA. Fluids are introduced to and drained out of the flow cell through integrated stainless steel tubing within the inserts. The bottom PMMA insert also features a plastic screen disc that acts as a support for a 3-μm Nuclepore polycarbonate, track-etch filter (Whatman, Florham Park, New Jersey, United States), which serves as a lymphocyte capture and red blood cell separation membrane. A gasket between the membrane and the top insert prevents leaks and ensures that the entire sample is delivered into the flow cell and filtered through the membrane. The top outlet is used with lateral flow for the removal of air bubbles.

**Figure 1:**
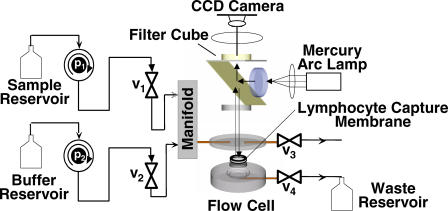
Components of the ETC System A fluid delivery system is used to introduce sample containing fluorescently stained lymphocytes in whole blood and wash buffer to a capture flow cell. Lymphocytes captured within the flow cell are visualized with a fluorescence imaging station using a mercury pressure lamp as a light source, and a CCD for image collection. Raw data images are then processed and analyzed using an automated algorithm run by an attached computer. The flow cell includes a polymer membrane supported on a chip and two transparent polymethylmethacrylate inserts that allow for the optical evaluation of captured lymphocytes.

### Fluid Delivery System

In initial studies, we used a single peristaltic pump to deliver sample and washes to the flow cell. Subsequently, a partially automated fluid delivery system was developed. This functional adaptation uses two miniature OEM peristaltic pumps, each in conjunction with a pinch valve, and 0.031-in. (0.79-mm) silicone tubing capable of delivering flow rates of 46–920 μl/min to the flow cell. Integrated software (LabVIEW, National Instruments, Austin, Texas, United States) directs delivery of whole blood samples and washes to the flow cell using the appropriate pumps and valves. Sample filtrate, including red blood cells, is captured in a waste reservoir.

### Optical Station and Image Capture

The flow cell was positioned on the stage of a modified BX2 Olympus (Tokyo, Japan) compound microscope equipped with a 10× objective lens and a high-pressure 100 W mercury burner arc lamp as a light source. Focusing was maintained on a fixed plane throughout the duration of the assay. Visualization of AlexaFluor-647-stained lymphocytes was achieved using a Cy5 filter cube (620 nm excitation, 660 nm long-pass beam splitter dichroic mirror, and 700 nm emission), while AlexaFluor-488-stained lymphocytes were visualized with a fluoroisothiocyanate (FITC) filter cube (480 nm excitation, 505 nm long-pass beam splitter dichroic mirror, and 535 ± 25 nm emission). For each study participant, images were obtained from each of five nonoverlapping regions of the lymphocyte capture membrane in the flow cell, using a 12-bit CCD digital camera (DVC, Austin, Texas, United States) mounted on the microscope. Each imaged region represented 0.18 μl of whole blood, so that for each assay, cells were counted from a total volume of 0.9 μl of blood. Each region was imaged serially with both filter cubes. The corresponding images were stored separately as monochromatic eight-bit images for subsequent digital image analysis and automated cell counting.

### Image Analysis

Images were analyzed using a custom algorithm supported by Image-Pro Plus (Media Cybernetics, Silver Spring, Maryland, United States) processing software. An iterative approach allowed for flexible analysis of data acquired under different conditions of illumination, focus, and sampling. For each iteration, an upper and lower value defined a range of green or red intensities that were then used to segment the image. Pixels whose intensity values fell within the defined range were reassigned values of one, while all others were set to zero. The process yielded a binary version of the original eight-bit image. A lymphocyte selection algorithm was then applied. Objects (i.e., lymphocytes) were defined as contiguous groups of pixels with values of one. Object selection was refined by a lymphocyte profile (defined by size, aspect ratio, and uniformity); objects not fitting the profile were not counted. The number of counted objects was recorded for each iteration. From one iteration to the next, the upper and lower intensity limits used to segment the image were both increased by a single intensity count. The final cell count per image was the maximum object count over 256 iterations (upper intensity limits 1→255) for which the average object roundness fell below a threshold value. In this manner, the software algorithm determined the optimal analysis parameters for each image individually, greatly relaxing the stringency of image capture requirements. Cell counts were recorded in a spreadsheet as numbers of CD4^+^CD3^−^, CD4^+^CD3^+^, CD4^−^CD3^+^, CD8^+^CD3^−^, CD8^+^CD3^+^, CD8^−^CD3^+^, and CD4^+^CD8^+^ cells, depending on the combination of antibodies used. Absolute CD4 counts were recorded as the summed number of CD4^+^CD3^+^ cells counted over five images, normalized per microliter of imaged blood. CD4:CD8 ratios were recoded as the ratio of CD4^+^CD3^+^ cells to CD8^+^CD3^+^ cells counted over five images. Relative CD4 abundance as a percentage of total T lymphocytes was recorded as 100 times the ratio of CD4^+^CD3^+^ cells to total CD3^+^ cells, with cells counted over five images.

### Lymphocyte Staining and Delivery

Antibodies utilized in these studies were stored at 4 °C and centrifuged to remove precipitated material prior to use. This process ensured removal of fluorescent particulate matter that could be captured by the membrane and might interfere with imaging. For the initial dilution control studies, CD4 cells were purified by immunomagnetic separation from donor buffy coats. CD4 cells labeled with AlexaFluor-488-conjugated anti-CD4 antibodies (A21335, clone 289–14120, Molecular Probes, Eugene, Oregon, United States) were introduced to the flow cell in amounts ranging from zero to 200,000 cells, and washed with phosphate buffered saline (PBS). For whole blood studies, 33 μl of whole blood collected by venipuncture was incubated at ambient temperature (20–25 °C) with 3 μl of AlexaFluor-488- and AlexaFluor-647-conjugated antibodies to CD4 and CD3 (A21332, clone 289–13801, Molecular Probes), respectively, and allowed to react for 8 min. Similarly, for CD8 enumeration, 33 μl of whole blood with 3 μl of AlexaFluor-488- and AlexaFluor-647-conjugated antibodies to CD8 (A21340, clone 289–13804, Molecular Probes) and CD3, respectively, was allowed to react for 8 min at ambient temperature. Stained blood samples were brought up to 1,000 μl with PBS, half of which was introduced directly into the flow cell (representing 16.5 μl of the original sample of blood) and then washed with 1 ml of PBS. Because red blood cells are mechanically separated from white blood cells, red blood cell lysis is not necessary. Images of labeled cells captured on the membrane were obtained and analyzed as described above. For SEM (scanning electron microscopy), a fixative (2% paraformaldehyde/2.5% glutaraldehyde) was added into the flow cell and rinsed with PBS. The filter was removed from the flow cell, fixed for 90 s with OsO_4_ vapor, and then dehydrated with EtOH/HMDS. The same SEM protocol was applied to a drop of whole blood on a glass slide.

### Study Participants and Comparison to Flow Cytometry

Blood was obtained from HIV-1-uninfected control participants and HIV-infected participants at the Massachusetts General Hospital in Boston, Massachusetts, United States, and from HIV-infected participants at the Botswana–Harvard AIDS Institute HIV Reference Laboratory in Gaborone, Botswana. The Botswana samples originated from a study of HIV-infected pregnant women attending maternal–child health clinics in Gaborone or three nearby villages, Molepolole, Mochudi, and Lobatse. Six infants were also included in the study. Three milliliters of venous whole blood was collected from each participant (in EDTA anticoagulant). All samples were run on the microchip on the day of blood collection. Parallel samples were processed using standard four-color flow cytometry on a Becton Dickinson FACSCalibur, using the MultiTEST reagents and TruCOUNT beads, and analyzed using MultiSET software. All samples were processed by flow cytometry according to standard operating procedure in the HIV reference laboratory in Botswana. The majority were processed within 24 h of blood collection, and all were processed and analyzed within 72 h of blood collection. A total of 70 participants were enrolled, including 64 adults and six infants. Three adults did not have flow cytometry results available, leaving 67 participants for analysis. The study was approved by the institutional review boards of the participating institutions. For a preliminary assessment of assay variability, blood from a single study participant was assayed as described above 20 separate times over the course of a single afternoon by a single operator.

### Statistical Methods

The accuracy of the microchip-based CD4 counting system was determined by comparing results directly to parallel samples processed by flow cytometry using Passing–Bablok regression analysis and the Bland–Altman methods comparisons approach [25,26]. For assay reproducibility, a coefficient of variance was calculated from 20 replicates of a single participant. Data were analyzed and processed using Analyse-It software (Analyse-It Software, Leeds, United Kingdom).

## Results

In initial experiments using the original ETC system [21–23], microspheres were coated with monoclonal antibodies to the lymphocyte surface markers CD3, CD4, or CD8, followed by microfluidic delivery of fluorescently labeled lymphocytes from whole blood obtained from non-HIV-infected participants. Although lymphocytes were readily captured, precise quantification of cell numbers and CD4 cell counts were not possible using the microsphere as a surface for lymphocyte capture (data not shown). We next modified the flow cells with a disposable, microporous membrane filter for lymphocyte capture. A single polycarbonate, track-etch membrane with 3-μm pores was immobilized and secured within the flow cell, creating a lymphocyte capture surface with a surface area of 80 mm^2^. Whole blood samples were delivered to the flow cell from a sample reservoir tube, and the membrane within the flow cell was washed with PBS from a second reservoir. As in the original ETC system, cells were imaged under fluorescence optics using a mercury arc lamp light source and a CCD camera (Figure 1).

To confirm that cells could be adequately captured, 33 μl of unprocessed whole blood from non-HIV-infected participants was incubated for 8 min with fluorophore-conjugated anti-CD4 antibodies, and delivered by a peristaltic pump to the modified microfluidics chip. Red blood cells passed readily through the pores under appropriate fluid flow conditions. In contrast, the majority of white blood cells were captured onto a single imaging focal plane (Figure 2). This mechanical separation of autofluorescent red blood cells allows for the imaging and counting of white blood cells from unprocessed whole blood without additional sample processing, such as centrifugation or red blood cell lysis. Using the digital imaging system originally developed for microsphere-based capture in the ETC system, fluorescently labeled white blood cells can then be imaged directly on the chip and counted.

**Figure 2:**
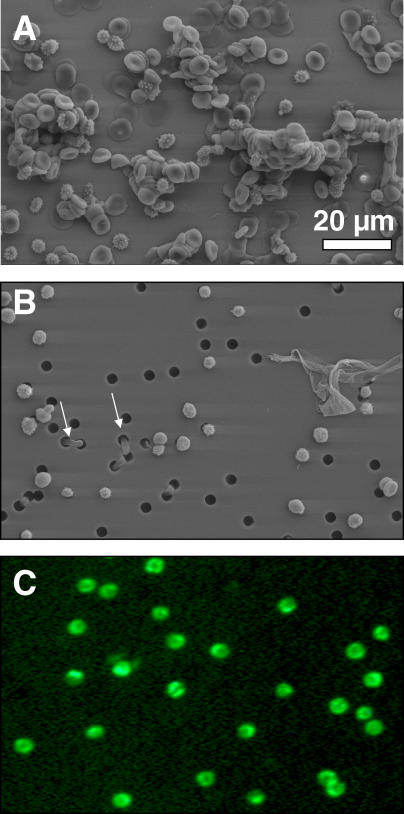
The Membrane Flow Cell Selectively Captures Lymphocytes and Provides for the Removal of Red Blood Cells without Sample Processing (A) A whole blood sample collected atop a glass slide and imaged by a scanning electron microscope reveals the overabundance of red blood cells in the sample. (B) A whole blood sample processed through the flow cell reveals that lymphocytes are captured on the membrane support while red blood cells are largely excluded from within the cell. Arrows indicate red blood cells passing through the membrane. (C) Fluorescent antibody stain specific for a lymphocyte marker is used to visualize captured lymphocytes within the flow cell in a representative single-color data image.

To assess the analytical validity of the membrane-based microchip system, we first performed a dilution control study to evaluate the correlation between total fluorescence intensity and the absolute number of purified CD4 cells from non-HIV-infected participants (labeled with fluorophore-conjugated anti-CD4 antibody) captured in the microchamber. The results show a linear correlation between the number of cells in the sample and the intensity of light emitted from the membrane filter (*R* ^2^ = 0.999) for a range of CD4 cell counts relevant to advanced HIV disease (0–200 CD4 cells/μl blood) (Figure 3). This dose–response study established proof of the concept that a modified microfluidic flow cell and a digital image analysis system can accurately detect and measure populations of whole blood lymphocytes labeled with fluorescent markers.

**Figure 3:**
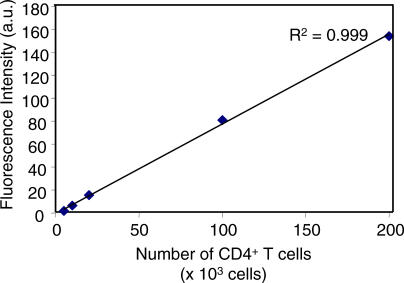
CD4 Lymphocyte Dose Response Purified CD4 cells were labeled with AlexaFluor-488-conjugated anti-CD4 antibodies, introduced to the flow cell in amounts ranging from zero to 200,000 cells and imaged. There is a linear correlation between the number of cells in the sample and the intensity of light emitted from the membrane filter (*R* ^2^ = 0.999).

We next quantified the percentages of CD3, CD4, and CD8 cells in whole blood samples from healthy control participants using this system. Prior to delivery to the flow cell, we labeled a 33-μl whole blood sample with 3 μl of fluorophore-conjugated anti-CD3 and anti-CD4 antibodies for 8 min off chip, then diluted the sample with 961 μl of PBS, and delivered 500 μl of the resulting sample (containing 16.5 μl of blood) to the flow cell using a fluidics controller. Digital images from one region of the lymphocyte capture membrane were obtained with two different emission filters, one specific for the AlexaFluor-488-conjugated antibody used to stain CD4^+^ T lymphocytes green (Figure 4A), and the other specific for the AlexaFluor-647-conjugated antibody used to stain CD3^+^ T lymphocytes red (Figure 4B). Automated digital merging of the two images and image processing allowed the system to distinguish the CD3^+^CD4^+^ T lymphocytes of interest (i.e., “CD4 cells”), which appear yellow, from the CD4^+^CD3^−^ monocytes (green) and the CD3^+^CD4^−^ T lymphocytes (red) (Figure 4C).

**Figure 4:**
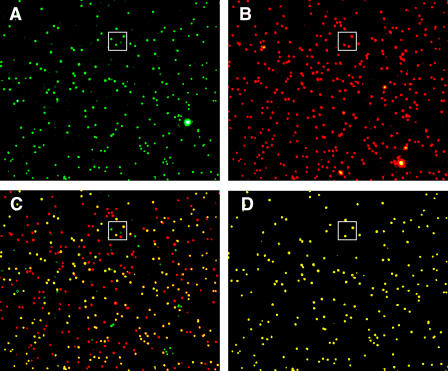
Data Collection and Processing for Digital Images Obtained from a Single Diluted Whole Blood Specimen from an HIV-Infected Participant A total of 16.5 μl of whole blood stained with antibodies specific for CD4 and CD3 markers is delivered to the flow cell after 8 min, and an image of the same region of the membrane is obtained with two different emission filters. (A) AlexaFluor-488-conjugated anti-CD4 antibody stains CD4^+^ cells (T lymphocytes and monocytes) green. (B) AlexaFluor-647-conjugated anti-CD3 antibody stains CD3^+^ T lymphocytes red. (C) By digitally merging the two images, CD3^+^CD4^+^ T lymphocytes (i.e., “CD4 cells”) appear yellow and are distinguished from CD4^+^CD3^−^ monocytes (green) and CD3^+^CD4^−^ T lymphocytes (red). (D) A lymphocyte selection algorithm is applied to the merged image, based on a lymphocyte profile as defined by size, shape, and uniformity. Objects not fitting the lymphocyte profile are deleted while remaining objects are selected and ultimately counted. A similar protocol to count CD8 cells is used in each participant. Boxed region indicates two CD4^+^ cells (yellow in [C]) in the original (A and B), merged (C), and processed (D) images. Large green and red objects seen in some images represent aggregates of fluorescent antibody.

We next developed a custom algorithm for translating these digital images into accurate CD4 and CD8 T cell counts using pixel analysis with the aid of a commercial image processing package. Automated counting of the three subsets of cells was based on object size, aspect ratio, and uniformity, iterated across the range of color intensity levels. As shown in Figure 4D, a binary mask first removes the unwanted cell types, and residual objects representing CD4 T cells are counted. A similar protocol was applied to a second aliquot of blood stained with AlexaFluor-647-conjugated CD3-specific antibody and AlexaFluor-488-conjugated CD8-specific antibody to visualize and count CD3^+^CD8^+^ T lymphocytes.

In order to calculate an absolute CD4 count with standard flow cytometry, one of two measures must be undertaken to calculate a concentration in cells per microliter. Either a standardized reference reagent, such as calibration beads at a known concentration, can be added to the assay (“single-platform” flow cytometry), or an absolute total lymphocyte count in cells per microliter can be obtained on a hematology analyzer (“dual-platform” flow cytometry). The microchip assay we describe here uses a direct volumetric method and functions as a single-platform approach. By delivering a consistent volume of blood to the flow chamber (16.5 μl of stained whole blood, diluted to a total volume of 500 μl of PBS), and calculating the unit volume of blood per digital image (0.18 μl), we were able to count the total number of CD4^+^CD3^+^ cells in 0.9 μl of blood, and determine the absolute CD4 count per microliter.

We next tested this rapid, whole blood microchip assay in a series of samples acquired in an HIV reference laboratory in Botswana. Seventy consecutive HIV-infected participants presenting to the HIV reference laboratory for standard CD4 counting as part of a vertical transmission study were enrolled, of whom 64 were adult women and six were infants. Parallel samples were processed by standard four-color flow cytometry on a Becton Dickinson FACSCalibur. The time from blood collection to complete analysis and results reporting using the chip-based assay was approximately 15 min per sample. Three adult participants did not have valid flow cytometry results available, leaving 61 adults and six infants for analysis.

Representative processed data images from three participants, two adult women and one infant, are shown in Figure 5. Figure 5A shows a 31-y-old woman with an absolute CD4 count by flow cytometry of 83 cells/μl. While numerous CD3^+^ T cells (red) are present as well as scattered monocytes (green), her low CD4 count is reflected in the few double-labeled CD3^+^CD4^+^ T cells (yellow) seen in the image. Similar representative data images from a young woman with a CD4 count of 271 cells/μl by flow cytometry and a 5-mo-old infant with a CD4 percentage of T lymphocytes of 0.39 by flow cytometry are also shown in Figure 5B and 5C, respectively. These images illustrate the dynamic range of the membrane capture and digital image analysis system, including the ability to quantify both absolute CD4 counts and CD4 percentages.

**Figure 5:**
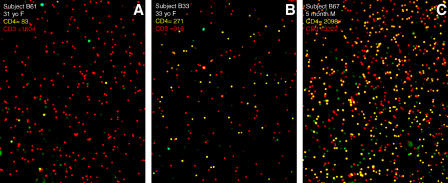
Representative Processed Data Images from Three Participants in Botswana The participants included (A) a 31-y-old woman with a CD4 count of 83 cells/μl by flow cytometry; (B) a 33-y-old woman with a CD4 count of 271 cells/μl by flow cytometry; and (C) a 5-mo-old infant with an absolute CD4 count of 2,098 cells/μl and a CD4:CD8 ratio of 1.80 by flow cytometry. In these images, CD3^+^CD8^+^ T cells appear red, monocytes appear green, and CD3^+^CD4^+^ T cells appear yellow. Each image reflects 0.18 μl of whole blood.

We compared results from our microchip assay with results available from flow cytometry, the latter obtained on a FACSCalibur through standard clinical laboratory operating procedures. The data for adult absolute CD4 counts are plotted in the Bland–Altman methods comparison plot shown in Figure 6. For 61 adult participants with CD4 counts ranging from 35 to 1,087 cells/μl (mean, 372 cells/μl) by flow cytometry, results show a good correlation between absolute CD4 counts measured by our microchip assay and those measured by flow cytometry. Bland–Altman methods comparison analysis shows a bias of −50 cells/μl (95% confidence interval, −81 to −20 cells/μl), and good 95% limits of agreement (Figure 6). Several of the results from participants at the higher end of absolute CD4 counts fall outside the 95% limits. For these participants, individual lymphocytes may overlap in the digital images (as seen in Figure 5C), which can interfere with the accuracy of the lymphocyte counting algorithm. In resource-limited settings, the primary use of CD4 counts is as a trigger to initiate antiretroviral therapy, which typically occurs at a CD4 count of 200 cells/μl. Higher CD4 count thresholds of 350 and 500 cells/μl are also used to increase the intensity of monitoring. For these values, the sensitivity and specificity of our method are: CD4 < 250, sensitivity = 0.86, specificity = 0.81; CD4 < 350, sensitivity = 0.97, specificity = 0.83; and CD4 < 500, sensitivity = 0.96, specificity = 0.85.

**Figure 6:**
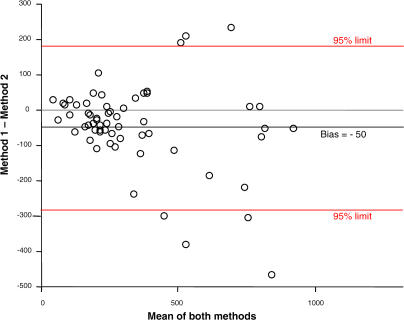
Methods Comparison and Correlation Studies for Absolute CD4 Counts in 61 Adults in Botswana Bland–Altman methods comparison plot comparing absolute CD4 cells per microliter of whole blood obtained by the microchip system as compared to standard four-color flow cytometry processed in parallel on a FACSCalibur in 61 HIV-infected adult participants. There is a proportional bias of −50 cells/μl relative to flow cytometry. Grey line indicates zero bias. Red lines indicate upper and lower 95% limits of agreement.

One important application of our method is in pediatric HIV monitoring. The wide range of normal absolute CD4 counts in infants and children requires the use of CD4:CD8 ratios or CD4 percentages in pediatric infection. Results for CD4:CD8 ratios and CD4 percentages of T lymphocytes for all 67 participants (61 adults and six infants) are shown in Figure 7. Agreement, bias, and correlations between the microchip method and flow cytometry are excellent for both CD4 percentages of T lymphocytes (Figure 7A and 7B) and CD4:CD8 ratios (Figure 7C and 7D). Bland–Altman plots for both CD4 percentages of T lymphocytes and CD4:CD8 ratios show low proportional bias, with tight 95% limits of agreement. Correlations are excellent for both CD4 percentages of T lymphocytes (*r* = 0.98, *p* < 0.0001) and CD4:CD8 ratios (*r* = 0.98, *p* < 0.0001). Overall, the data show that all three approaches to measuring CD4 cell counts can be accurately quantified using the microchip method, and that both adult and pediatric CD4 results can be obtained.

**Figure 7:**
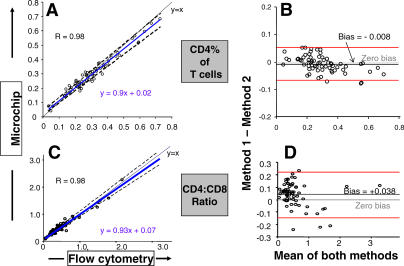
Methods Comparison and Correlation Studies for CD4 Percentages of Total T Cells and CD4:CD8 Ratios in 67 Human Subjects (A and B) CD4 percentages of total T cells and (C and D) CD4:CD8 ratios in 67 human participants, including 61 adults and six children. In Passing–Bablok correlation plots (A and C), solid black lines indicate identity, blue lines indicate the observed correlations, and dashed black lines indicate 95% confidence limits. Correlations for CD4 percentages of total T cells (*r* = 0.98, *p* < 0.0001) and CD4:CD8 ratios (*r* = 0.98, *p* < 0.0001) are shown. For Bland–Altman methods comparison plots (B and D), notations are as described in Figure 6 caption.

To determine assay variability, we examined 20 replicate samples of blood from a single participant over the course of one day, using the established basic protocol. We determined that the coefficient of variance was 12% (data not shown), which is similar to other methods of CD4 counting [27]. Although the assay described here introduced 16.5 μl of blood into the system, the actual volume of blood analyzed by digital image analysis is only 0.90 μl. We have conducted preliminary studies that suggest that we can accurately measure CD4 counts from less than 5 μl of blood obtained via fingerstick (data not shown); additional studies will be required to assess the correlation between CD4 counts obtained by fingerstick and by venipuncture.

## Discussion

Our results provide proof of principle that low-cost microfluidic structures combined with fluorescence imaging and digital image analysis can be successfully applied to the measurement of CD4 cell counts, which are critical to the clinical management of HIV infection. The method described here can deliver both absolute CD4 counts for adult monitoring, and CD4 percentages or CD4:CD8 ratios for pediatric monitoring. Most importantly, the rapid and accurate CD4 assessments obtained with this method, together with its anticipated low cost relative to flow cytometry, may make this type of approach ideal for resource-scarce settings. As our results show, this method may be less accurate at the higher range of CD4 counts, where cells may be more likely to overlap in our digital images. While this may limit its applicability, our method is accurate at CD4 counts below 500 cells/μl, which represent the clinically relevant CD4 levels in resource-poor settings. In addition, both the bias in the method described here (−50 cells) and the accuracy at higher CD4 counts are likely to be improved significantly by the further development of a disposable microfluidic cartridge, where the volume of distribution of the sample will be much smaller, and more accurate volumetric control will be possible.

Our study was designed to evaluate the accuracy of our method against the gold standard in a population of adults. During enrollment, a small number of pediatric samples were made available to us by the staff at Princess Marina Hospital in Botswana. We chose to include these samples in the data presented here to provide proof of principle that pediatric CD4 percentages can also be assessed with this method. Although only six pediatric samples were available, limiting claims of statistical significance, we believe the issue of pediatric CD4 count monitoring to be of such importance that the data merited inclusion. Excluding the six pediatric samples does not affect the analysis.

The results presented here were obtained with a stationary, tabletop monitoring system using a standard epifluorescence microscope and commercial image processing software. While the methods we described provide the basis for a highly portable and flexible miniaturized CD4 counting system, it should be emphasized that a number of additional developments are required to enable the widespread use of this approach in resource-limited settings. With additional engineering of optics, electronics, and mechanical components along with advancements in integrated microfluidic systems, it should be possible to develop a point-of-care instrument that is battery-powered, uses simple light emitting diodes (LEDs), and secures analyzable digital images with affordable video imaging chips. When combined with an embedded microprocessor and disposable assay cartridges for both adult and pediatric monitoring manufactured from injection-molded plastic, it should be possible to create a functional CD4 counting device that can be used at the point of care. Further trials in a larger, more diverse cohort of patients, including adult men and children, will be necessary to confirm the accuracy of the method, including an assessment of assay bias and reproducibility. Such a device is currently in commercial development, and may be available by early 2006. While it is too early to provide an accurate cost estimate for a portable instrument and disposable plastic CD4 assay, we expect the equipment cost would be substantially lower than for flow cytometry, and the assay cost would be similar to assays using existing methods (Table 1).

**Table 1:**
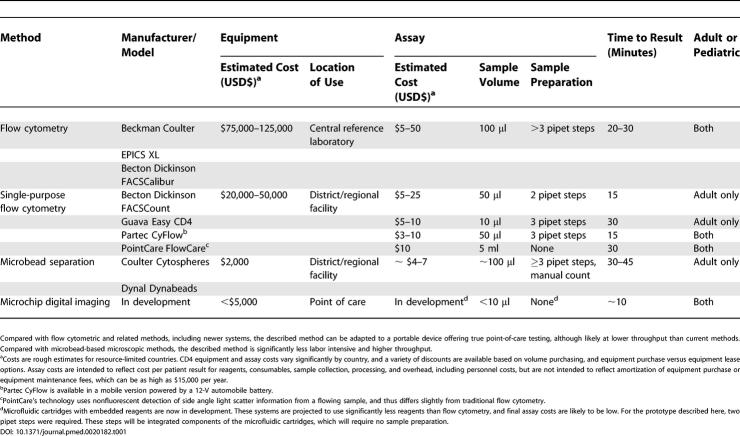
A Comparison of Methods for CD4 Determination

Although several CD4 counting systems are now used in resource-limited settings, they remain suboptimal to meet the needs of HIV care and treatment scale-up. None can truly be used at the point of care beyond a district hospital or similar facility, and either the capital and operating costs remain high, or throughput is low, or both (Table 1). Pediatric monitoring using CD4 percentages also remains largely unavailable. The method we describe here addresses several of the limitations of performing diagnostic assays in resource-limited settings. First, sample volumes are minimal, so that tests can be performed on fingerstick samples of blood, circumventing the need for venipuncture, and minimizing both medical waste and operator exposure to biohazardous material. Second, reagent use is minimized in the microchip system, reducing reagent costs by as much as 90%. Third, labor- and equipment-intensive sample preparation is eliminated. Fourth, the microchip CD4 assay is extremely rapid. CD4 results in the prototype system described here are available in less than 15 min from the time of blood collection. In a mature microfluidic device with push-button operation, results should be available in less than 10 min, and thus can be used to make real-time clinical decisions at the point of care. Fifth, the assay is technically simple, analogous to a portable glucometer, and ultimately will be useable by a health-care worker in remote settings with minimal training, extending the reach of CD4 assays to district hospitals and remote clinics, and reducing labor costs. Sixth, both adult and pediatric monitoring are possible.

We believe that the future of low-cost diagnostics for use in the developing world lies in the development of new lab-on-a-chip technologies that integrate sample preparation and sample measurement systems into miniaturized devices with minimal power requirements. Preliminary cost estimates for the instrumentation here described suggest, at a minimum, a 10-fold reduction in the cost for the associated measurement system. Further, reagent consumption for the microchip system can be reduced by a similar factor relative to flow cytometry, while sample storage and shipping costs are expected to be reduced dramatically by virtue of the point-of-care capabilities of this new lab-on-a-chip structure. The importance of microtechnologies to the realities of laboratory infrastructure worldwide has been recognized previously [28–30]. Although CD4 counting represents the most urgent need in HIV diagnostics for resource-poor settings, the microchip platform is adaptable to other important assays. Through the interface of the lymphocyte capture membrane described here with the previously reported microchip arrays, cellular assays like CD4 counts can be multiplexed with other molecular biomarker measurements (i.e., proteins and nucleic acids) on a single miniaturized chip. The rapid extension of the chip-based CD4 counting method described here to HIV RNA measurements, diagnostics for opportunistic infections, liver enzymes, and other biochemical markers of interest in infectious disease is feasible.

**Patient Summary**

**Background**

Most HIV-infected people don't develop AIDS right away, because their immune systems can keep the virus in check for months and sometimes years. In general, doctors don't recommend that infected people start taking HIV medications while their immune system is still healthy. Doctors know whether or not a patient's immune system is healthy-and therefore whether or not to start treatment-by measuring the "CD4 count." This is the number of CD4 cells in a sample of blood. CD4 cells, also called CD4+ T cells, are a type of white blood cell that fights infection. HIV destroys CD4 cells, weakening the body's immune system and ultimately causing AIDS. CD4 counts should be determined before a patient receives antiretroviral therapy and then measured regularly while the patient is on therapy.

**Why Was This Study Done?**

Most tools available to count CD4 cells are large and expensive to buy, and every actual count is also expensive and difficult to carry out. These tools are therefore unsuitable for many low-income countries. The researchers wanted to develop a tool that allows easier and cheaper measurement of CD4 cells, and is small and simple enough that health-care workers can take it to patients when they visit them in remote areas.

**What Did the Researchers Do?**

They built a prototype for a new tool that counts CD4 cells in a simpler and cheaper way. They then took samples from 61 adults and six children and compared the results when they used both the standard technologies and their prototype counter.

**What Did They Find?**

They found that their prototype works well and is indeed cheaper and easier to use. It also appears to be just as reliable as the large and more complicated machines in helping doctors make decisions about when to start therapy and when to change therapies. They have done enough samples from adults infected with HIV to be confident about that. It looks like it might work for infected children as well, but they haven't done enough child samples yet to be certain.

**What Does This Mean?**

This suggests that with some additional work, it should be possible to develop a handheld CD4 counter that is cheap, easy, and transportable. This could make a big difference for the care of HIV patients in developing countries and other remote areas.

**What Next?**

There is still more development work to do to get from the prototype to a handheld counter, and the researchers should also study more samples from children to see whether the new test is equally reliable for pediatric patients.

**Additional Online Resources**

Information about CD4 monitoring for HIV/AIDS patients can be found at the following Web sites.

World Health Organization (search for CD4 count):

http://www.who.int/en/

The World Health Organization's specific review of CD4 counting technologies:

http://www.who.int/3by5/amds/Suzanne_Crowe.pdf

The Well Project:

http://www.thewellproject.org/

The Well Project's specific page on CD4 counts:

http://www.thewellproject.org/Treatment_and_Trials/First_Things_First/Understanding_CD4_and_CD8_Cells.jsp

Find Diagnostics, an organization focused on affordable diagnostics for infectious diseases worldwide:

http://www.finddiagnostics.org/

The Forum for Collaborative HIV Research, a consortium with an advocacy and funding role for HIV/AIDS research priorities, including low-cost CD4 and viral load tests:

http://www.hivforum.org/

Project Inform (search for CD4 and monitoring):

http://www.projectinform.org/

## Abbreviations

CCD: charge-coupled device
ETC: electronic taste chip
PBS: phosphate buffered saline
